# Unveiling the seasonal dynamics of leishmaniosis: High-resolution monthly potential risk mapping and future expansion of *Leishmania infantum* in Europe

**DOI:** 10.1371/journal.pntd.0014673

**Published:** 2026-09-16

**Authors:** Elena Infante González-Mohino, Iván Rodríguez-Escolar, Alfonso Balmori-de la Puente, Ione Urizar-Molina, Alberto Gil-Abad, Manuel Collado-Cuadrado, Alessia Crippa, Rodrigo Morchón

**Affiliations:** 1 Zoonotic Diseases and One Health Group, Faculty of Pharmacy, Centre for Environmental Studies and Rural Dynamization (CEADIR), University of Salamanca, Salamanca, Spain; 2 Ceva Santé Animale, Libourne, France; 3 Biomedical Research Institute of Salamanca (IBSAL), University of Salamanca, Salamanca, Spain; University of Queensland School of Veterinary Science, AUSTRALIA

## Abstract

**Background:**

Zoonotic leishmaniosis, caused by *Leishmania infantum*, is a major public health and veterinary concern in Europe. Traditionally restricted to the Mediterranean basin, the disease is currently undergoing a rapid geographical expansion towards Central and Northern Europe, driven by climate change and the plasticity of its vectors. Current epidemiological surveillance often relies on static annual prevalence maps, which fail to capture the critical intra-annual fluctuations in transmission risk. This study aims to bridge this gap by developing the first high-resolution spatiotemporal analysis of *L. infantum* potential infection risk across the entire European continent, providing a dynamic tool to optimize control strategies under a One Health framework.

**Methods:**

We developed Ecological Niche Models (MaxEnt) for the two primary European vectors, *Phlebotomus perniciosus* and *Phlebotomus perfiliewi*. To estimate the transmission capability, we integrated these habitat suitability models with a biological model of parasite development (Rioux’s algorithm), which calculates the infection rate based on daily mean temperatures (10–30 °C thermal window). This integration allowed for the generation of continuous annual and monthly potential risk maps. Furthermore, we projected the potential infection risk to the year 2100 under the SSP5-8.5 climate change scenario to quantify future range shifts and potential expansion into non-endemic territories.

**Results:**

The models exhibited excellent predictive performance (AUC ≥ 0.900), with annual mean temperature being the dominant driver for both vectors. The results confirmed high endemic risk in the Mediterranean basin but revealed significant emerging risk zones in the Balkans, Romania and the Southern Plateau of Spain. The monthly analysis unveiled a dynamic seasonality: transmission is latent in winter, restricted to southern coastal refuges, but expands latitudinally during spring and summer, covering most of Central Europe before retracting in autumn. Future projections for 2100 indicate an alarming 347.9% net increase in infection risk areas. The models predict a massive colonization of northeastern Europe and high-altitude regions, with no current risk areas becoming unsuitable.

**Conclusions/Significance:**

This study demonstrates that *L. infantum* transmission in Europe is a highly dynamic phenomenon defined by seasonal thermal windows rather than static boundaries. The projected expansion towards northern latitudes challenges the historical notion of leishmaniosis as solely a southern European disease. These dynamic risk maps represent a strategic One Health tool, enabling health authorities and veterinarians to transition from rigid calendar-based prophylaxis to adaptive, evidence-based interventions. This is particularly crucial for identifying transmission periods in emerging transition zones, ensuring better protection for both animal and human populations in a changing climate.

## Author summary

Zoonotic leishmaniosis, caused by the parasite *Leishmania infantum*, is an endemic disease in southern Europe that severely affects dogs and can be transmitted to humans by the competent vector. Historically, the risk was limited to the Mediterranean basin; however, climate changes and pet movements are facilitating the expansion of sand flies (the vector insect) toward the center and north of the continent. Until now, most risk maps were static, merely indicating whether an area was dangerous on an annual level, thus ignoring the fact that transmission risk fluctuates drastically depending on the season. In this study, we have developed the first high-resolution potential risk maps for the entire European continent that detail the probability of infection month by month. To achieve this, we employed mathematical models combining the distribution of the two primary vectors (*Ph. perniciosus* and *Ph. perfiliewi*) with the temperature required for the pathogen to develop inside the vector. Our results demonstrate that leishmaniosis transmission in Europe is a highly dynamic phenomenon: while the potential risk is negligible in winter (except on warm southern coasts), it expands rapidly during summer toward northern latitudes and mountain areas previously considered safe. Furthermore, our projections indicate that if global warming continues, the potential risk area in Europe could increase by nearly 350% by the year 2100. These findings are crucial for veterinarians and health authorities, as they provide a precise tool to determine not only *where* but *when* to apply preventive measures on dogs (such as repellent parasiticides and vaccines), thereby optimizing disease control under a One Health strategy.

## Introduction

Leishmaniosisrepresents one of the most complex and relevant vector-borne diseases in the context of public and veterinary health in Europe, with *Leishmania infantum* being the causative agent of zoonotic visceral leishmaniosis in the region [1,2]. The epidemiology of this parasitic disease cannot be dissociated from its main domestic reservoir: the dog. However, within a One Health framework, the role of the sylvatic cycle cannot be overlooked, as wild reservoirs such as foxes, lagomorphs or rodents also play a crucial role in maintaining and spreading the parasite in the European epidemiological context [2]. Canine leishmaniosis is not only a severe and potentially lethal disease for the animal, but prevalence in the canine population acts as the primary risk indicator for human infection. The presence of infected dogs, alongside these wild hosts, competent vectors, and a susceptible human population, creates the perfect scenario for zoonotic transmission of the disease under a One Health framework [3,4].

Historically, canine leishmaniosis in Europe was considered restricted to the Mediterranean basin and neighboring North African countries – where sand fly vectors find optimal ecological conditions – with a mean prevalence of 16.12% [5]. However, the European epidemiological scenario has undergone a drastic transformation over the last two decades. Central and Northern European countries, traditionally considered infection-free, are increasingly reporting cases [6–8].

Climate change is one of the main drivers of this spatial alteration, alongside pet travel and the vector expansion across the European continent [9–11]. Rising mean winter temperatures and prolonged summers are allowing sand flies to colonize previously sub-optimal ecological niches at higher latitudes [8]. Furthermore, a seasonal temporal shift is occurring and widening year by year. While transmission in southern Europe may be nearly perennial in certain warm foci, seasonal thermal windows are beginning to open in Central Europe, allowing the pathogen to complete its cycle within the vector [12].

To address this spatial complexity, Ecological Niche Models (ENMs) and machine learning algorithms such as MaxEnt have been consolidated as robust predictive tools for epidemiological surveillance, enabling the mapping of sand fly habitat suitability in regions such as Iran, Brazil, and Nepal [13–18]. In turn, recent studies have successfully mapped climatic suitability for *L. infantum* and its vectors on a continental European scale, identifying expansion trends toward Northern and Eastern Europe [6,19]. Additionally, detailed annual risk models have been developed for specific endemic countries like Spain, Portugal, and Greece, integrating biological factors such as the 10–30 °C thermal range for pathogen development within the vector [20,21]. However, these maps lack the high temporal resolution necessary to capture intra-annual transmission dynamics. In this regard, very recent investigations have begun to address this dynamic through monthly analyses, successfully defining the presence or absence of the vector activity period, as in the case of *Phlebotomus papatasi* in Iran [22]. Despite this advance in temporal resolution, there remains a need for models that go a step beyond vector activity and integrate a continuous assessment of infection risk covering the entire European continent, thus allowing for the precise identification of the highest-risk months in transition zones.

The main objective of this study was to analyze the monthly and annual potential risk of *L. infantum* infection for all of Europe by integrating large vector presence datasets with high-temporal-resolution bioclimatic variables and parasite development within the vector, providing a dynamic predictive tool that serves as a basis for European health authorities and clinical veterinarians in the implementation of prophylactic measures under the One Health concept.

## Methods

### Spatial modeling domain

**Fig 1:**
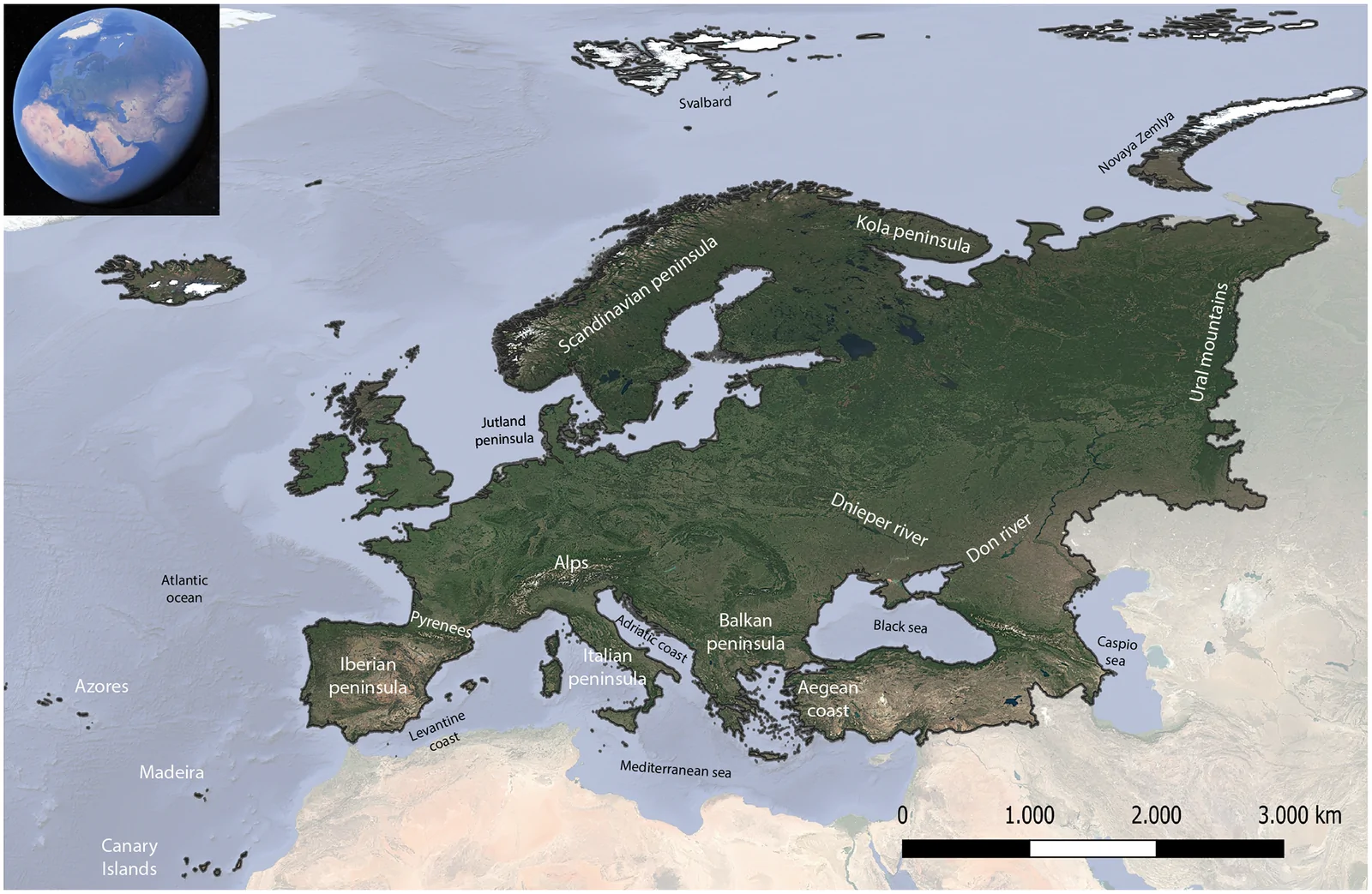
Satellite view of Europe illustrating the domain used for the spatial analysis. Basemap source: Esri World Imagery (Esri, Maxar, Earthstar Geographics, and the GIS User Community). Map generated in QGIS (version 3.34.15). Satellite base map: Map data 2026 ESRI. Boundary shapefiles obtained from GADM.

The spatial domain used to generate the models comprises the entire European continent, located in the Northern Hemisphere, bordered by the Arctic Ocean to the north, the Atlantic Ocean to the west, and the Mediterranean Sea to the south. Covering an area of approximately 10 million km^2^, Europe lies immediately north of Africa and borders the Asian continent to the east (Fig 1). Topographically, the study area ranges from extensive low-altitude plains to major mountain systems such as the Alps in Central Europe, the Pyrenees on the Franco-Spanish border, and the Urals as the eastern boundary. Its complex hydrographic network is defined by large basins that structure the territory, notably the Volga on the Russian side and the Danube-Rhine axis in Central Europe. Additionally, other river courses of regional relevance, such as the Thames (England), the Seine (France), the Ebro (Spain), and the Po (Italy), among others, are determinants [23].

According to the Köppen-Geiger classification, Europe is defined by great environmental diversity, transitioning from constant humidity to summer aridity. In the center and east of the continent, a continental climate (Df) predominates, with marked seasonality, harsh winters, and cool summers. Conversely, the southern basin is governed by a Mediterranean pattern (Csa and Csb), characterized by dry, hot summers with rainfall concentrated in the intermediate seasons. Towards the western edge, the influence of the Atlantic Ocean moderates temperatures, resulting in an oceanic climate (Cfb) with well-distributed precipitation throughout the year. Moving towards northern latitudes, this regime gives way to a subarctic climate (Dfc), defined by long, snowy winters, reaching tundra conditions (ET) in Iceland and the far north, where summer heat is almost nonexistent. Finally, the archipelagos of the Canary Islands, Azores, and Madeira introduce a subtropical component (temperate Csb with dry summer), enjoying mild and stable temperatures year-round, albeit with local hydrological variations dictated by their volcanic orography [24].

### Vector occurrence data

To generate habitat suitability models, georeferenced records of *Ph. perniciosus* and *Ph. perfiliewi* were collected. The selection of these two vector species was based on their high epidemiological relevance, as they are two of the most abundant and widely distributed species in the European territory [25,26]. Occurrence data were obtained through the European Network for Medical and Veterinary Entomology [27], under the supervision of the European Centre for Disease Prevention and Control [26], also integrating validated records from previous scientific studies [28]. A total of 1,456 georeferenced points for *Ph. perniciosus* and 1,384 points for *Ph. perfiliewi* were obtained. To ensure the quality of the biotic database, a cleaning protocol was applied, consisting of the removal of duplicate records and entries presenting inconsistencies or missing geographical coordinates. Once cleaned, the data were processed independently for each species using QGIS software (version 3.34.15). To mitigate potential biases derived from spatial autocorrelation and avoid overrepresentation in areas with greater sampling effort, spatial filtering was employed using a 1 km^2^ grid. Under this criterion, only one observation per cell was selected, ensuring that the sample distribution was representative of environmental heterogeneity. Following this refinement process, a final database of 1,204 points for *Ph. perniciosus* and 1,115 points for *Ph. perfiliewi* was consolidated, constituting the biotic basis for the development of the suitability models (S1 Fig).

### Bioclimatic and environmental variables

To obtain bioclimatic informations, 19 variables related to temperature and precipitation were downloaded from WorldClim 2.1, all with a spatial resolution of 1 km^2^ and representing contemporary climatic conditions [29]. To avoid information redundancy and ensure the statistical robustness of the models, multicollinearity was evaluated using R software (version 4.4.2) [30]. Pearson’s correlation coefficient (*r*) was calculated between all pairs of variables, excluding those with a significant correlation of |r| > 0.8. Following this process, seven final variables were selected for modeling: BIO_1_ (Annual Mean Temperature), BIO_2_ (Mean Diurnal Range: Mean of monthly temperature ranges), BIO_3_ (Isothermality: BIO_2_/BIO_7_ × 100, where BIO_7_ is the Temperature Annual Range), BIO_4_ (Temperature Seasonality), BIO_12_ (Annual Precipitation), BIO_15_ (Precipitation Seasonality: Coefficient of Variation), and BIO_17_ (Precipitation of Driest Quarter). Complementarily, variables related to landscape structure and anthropogenic pressure were incorporated. Data on shrub and herbaceous vegetation density were obtained from the EarthEnv platform [31], while the impact of human activity was integrated using the Human Footprint Index [32]. The latter indicator synthesizes data on built environments, population density, electric infrastructure, land use (croplands and pasture), and transport networks (roads, railways, and navigable waterways). Finally, all environmental layers underwent a spatial standardization process in QGIS. This procedure ensured that all predictors shared a uniform extent (European continent), the same pixel resolution (1 km^2^), and the EPSG:4326 (WGS 84) coordinate reference system, thus guaranteeing the geometric coherence necessary for modeling.

### Modeling approaches

Habitat suitability modeling for both vectors was performed using the MaxEnt algorithm [33] implemented via the kuenm package in R, which integrates calibration, evaluation, and final selection phases [34]. This method estimates relative environmental suitability based on the principle of maximum entropy, relating biotic records to environmental predictors and 10,000 background points (pseudo-absences) randomly distributed across the study area. For each species, 119 candidate models were evaluated, resulting from combining 17 regularization multipliers (ranging from 0.1 to 10) and different feature classes (linear, quadratic, and product). The selection of the final model was based on hierarchical criteria prioritizing statistical significance via Partial ROC (p < 0.05), an omission rate (OR) ≤ 5% to ensure predictive power, and the corrected Akaike Information Criterion (ΔAICc ≤ 2) to optimize complexity and avoid overfitting. Additionally, general discrimination capacity was validated using the Area Under the Curve (AUC), finally selecting the models that demonstrated the best balance between statistical fit and parsimony. It is important to note that the logistic output of MaxEnt reflects relative suitability rather than the absolute probability of presence, as the model is calibrated using presence-background data.

Using the Raster Calculator tool in QGIS (v. 3.34.15), an exploratory weighting was performed, and seven hypothetical scenarios were generated by combining both *Ph. perniciosus* and *Ph. perfiliewi* niche models in different contribution proportions (100:0, 90:10, 75:25, 50:50, 25:75, 10:90, and 0:100), applying a formula adapted from Balmori-de la Puente et al. [35]:


$$\begin{array}{c} Final\;MNE\;=\;\frac{{MNE}_{pn}*Pn+\;{MNE}_{pf}*Pf}{\text{1}\text{0}\text{0}} \end{array}$$

where *MNEpn* and *MNEpf* represent the habitat suitability models for *Ph. perniciosus* and *Ph. perfiliewi*, respectively, and *Pn*-*Pf* refers to the proportion of contribution (%) of each vector species to the model. Given that the MaxEnt algorithm is based strictly on presence and background data and cannot inherently incorporate continuous parameters such as vector abundance, competition or natural infection rates, these variations were designed as exploratory scenarios to simulate how different spatial proportions of relative suitability between the two vectors might influence the combined suitability of the vectors at a continental scale.

### Leishmania infantum infection rate

The infection rate, defined as the percentage of sand flies infected by *L. infantum*, was determined by adapting the biological model proposed by Rioux et al. [36] for *Ph. perniciosus* and *Ph. perfiliewi*. The calculation was based on the application of the following increasing logarithmic function:


$$\begin{array}{c} y=0,718[1-e^{\left(-0,237\cdot (x-8\right)}] \end{array}$$

where *y* represents the percentage of infected vectors and *x* represents the mean temperature. This relationship allows for the estimation of the infection rate within the critical range of 10–30 °C, a thermal interval that conditions the survival and replication of the parasite within the vector [36].

For this study, an R script based on the previous methodology of Rodríguez-Escolar et al. [20,21] was adapted and optimized through the implementation of an iterative loop. This script processed daily mean temperature data from the CHELSA database [37], allowing for the retrieval of not only the annual average but also monthly infectivity rates, which were incorporated into the analysis to better capture variation throughout the year. This approach represents an evolution from the method used in our previous studies [20,21], transitioning from a calculation based directly on static annual averages to a dynamic model capable of reflecting risk fluctuations throughout the year.

### Maps of annual and monthly potential risk of infection by Leishmania infantum and their validation

The habitat suitability models for *Ph. perniciosus* and *Ph. perfiliewi* with different contributions were, in turn, weighted with the *L. infantum* infection rate in Europe. Thus, seven different potential annual risk maps were generated.

To determine a single representative map with the highest fidelity to epidemiological reality, a validation and selection process was carried out. For this purpose, a robust validation dataset comprising 5,315 georeferenced records of veterinary-confirmed *L. infantum*-infected dogs across Europe was compiled. These data combined clinical veterinary records provided by the pharmaceutical company Ceva Santé Animale [38] and seroprevalence records from previous peer-reviewed studies [39–41]. Risk data were classified into five categories (’Very high,’ ‘High,’ ‘Medium,’ ‘Low,’ and ‘Very low’) using the Natural Jenks algorithm. This optimization method was applied independently to each scenario to capture the specific ecological discontinuities and natural breaks inherent to each vector combination, avoiding the data compression and bias associated with arbitrary fixed scales. Georeferenced points of positive dogs were overlaid on the seven generated maps, selecting as the definitive map the one that demonstrated the highest predictive capacity by concentrating the highest proportion of cases in the highest risk categories.

Once the *L. infantum* infection risk map with the optimal vector proportion was validated and selected, this combination was used to weight the generated monthly infectivity rates, thus obtaining the monthly risk maps.

### Future projections

To assess the impact of climate change towards the end of the century, exploratory habitat suitability projections for both sand fly species were projected in MaxEnt, using the previously selected parameters, to the 2100 time horizon (period 2081–2100). These projections integrated bioclimatic variables under the SSP5-8.5 scenario, representing a high CO_2_ emission trajectory to explore a worst-case climate change scenario in Europe [42], using data from the General Circulation Model (GCM) HadGEM3-GC31-LL [43]. This model was selected for its demonstrated ability to accurately capture the complex climatic patterns of the European continent and its extensive validation in ecological niche modeling studies [44,45]. Unlike the analysis of the current scenario, the infectivity rate for the year 2100 was calculated exclusively from the annual mean temperature (BIO_1_). This methodological simplification is necessary due to the absence of future daily mean temperature climate projections with the required resolution, which introduces a limitation for direct comparability due to these differing temporal resolutions. To partially address this constraint and capture potential shifts in the seasonal transmission envelope, the 2100 infectivity rates were additionally calculated using the bioclimatic variables BIO_10_ (mean temperature of the warmest quarter) and BIO_11_ (mean temperature of the coldest quarter).

To quantify the impact of climate changes on disease distribution, both the current potential risk map and the ones projected for 2100 were transformed into binary models. For this conversion, the 10th percentile training presence logistic threshold of the current model was applied, thus defining areas of potential risk. Finally, the biomod2 package in R was used to perform a range change analysis, calculating the percentage of pixels representing gains, losses, or persistence of *L. infantum* potential infection risk under the new climate scenario [46].

## Results

### Habitat suitability models of *Phlebotomus perniciosus* and *Phlebotomus perfiliewi*

Of the 119 models generated for each vector, those presenting the best fit according to the *kuenm* criteria were selected. For *Ph. perniciosus*, the optimal model was M_0.1_F_lqp, with an AUC of 0.900, while for *Ph. perfiliewi*, the model M_0.2_F_lqp was selected, reaching an AUC of 0.917 (S2 Fig). Habitat suitability for *Ph. perniciosus* recorded a maximum value of 0.9 (high suitability) and a minimum of 0.00006 (low suitability). The areas with the highest suitability were concentrated in the central-eastern and northwestern Iberian Peninsula, the Po Valley in Italy, the French Riviera, the Adriatic coast, and a large part of the Balkan Peninsula, extending to Armenia and Moldova. In contrast, Scandinavian countries, Russia, the United Kingdom, and Iceland presented low suitability levels. Regarding the model for *Ph. perfiliewi*, it reached a maximum value of 0.9 (high suitability) and a minimum of 0.00004 (low suitability). The regions of highest suitability for this species included the Po Valley, the French Riviera, the Adriatic coast, and the Balkans, also highlighting the Krasnodar region in Russia, the Caucasus (Armenia, Georgia, and Azerbaijan), and Moldova, along with medium suitability in the Ebro Depression in Spain. As in the previous case, northern European regions and the United Kingdom showed low habitat suitability.

Regarding the contribution percentages (Table 1), the annual mean temperature (BIO_1_) proved to be the determining factor in both cases, representing 43.30% for *Ph. perniciosus* and 39.76% for *Ph. perfiliewi*. In the *Ph. perniciosus* model, the next variables with the highest weight were the mean diurnal range (BIO_2_, 14.37%) and the human footprint (11.92%). For *Ph. perfiliewi*, temperature seasonality (BIO_4_, 12.71%) and mean diurnal range (12.38%) showed similar relevance after mean temperature. In both models, herbaceous density was the variable with the lowest explanatory capacity, with values below 0.80%.

**Table 1:** Percent contribution of the variables selected in the ecological niche model for *Phlebotomus perniciosus* and *Ph. perfiliewi.*

	Percentage contribution	
Variable	*Phlebotomus perniciosus*	*Phlebotomus perfiliewi*
Annual mean temperature (BIO_1_)	43.30%	39.76%
Mean Diurnal Range (BIO_2_)	14.37%	12.38%
Human footprint	11.92%	11.25%
Temperature Seasonality (BIO_4_)	7.99%	12.71%
Precipitation of Driest Quarter (BIO_17_)	7.69%	7.17%
Precipitation Seasonality (BIO_15_)	6.29%	4.81%
Isothermality (BIO_3_)	5.11%	7.20%
Annual Precipitation (BIO_12_)	1.45%	1.51%
Shrub density	1.18%	2.41%
Herbaceous density	0.70%	0.80%

### *Leishmania infantum* infection rate

**Fig 2:**
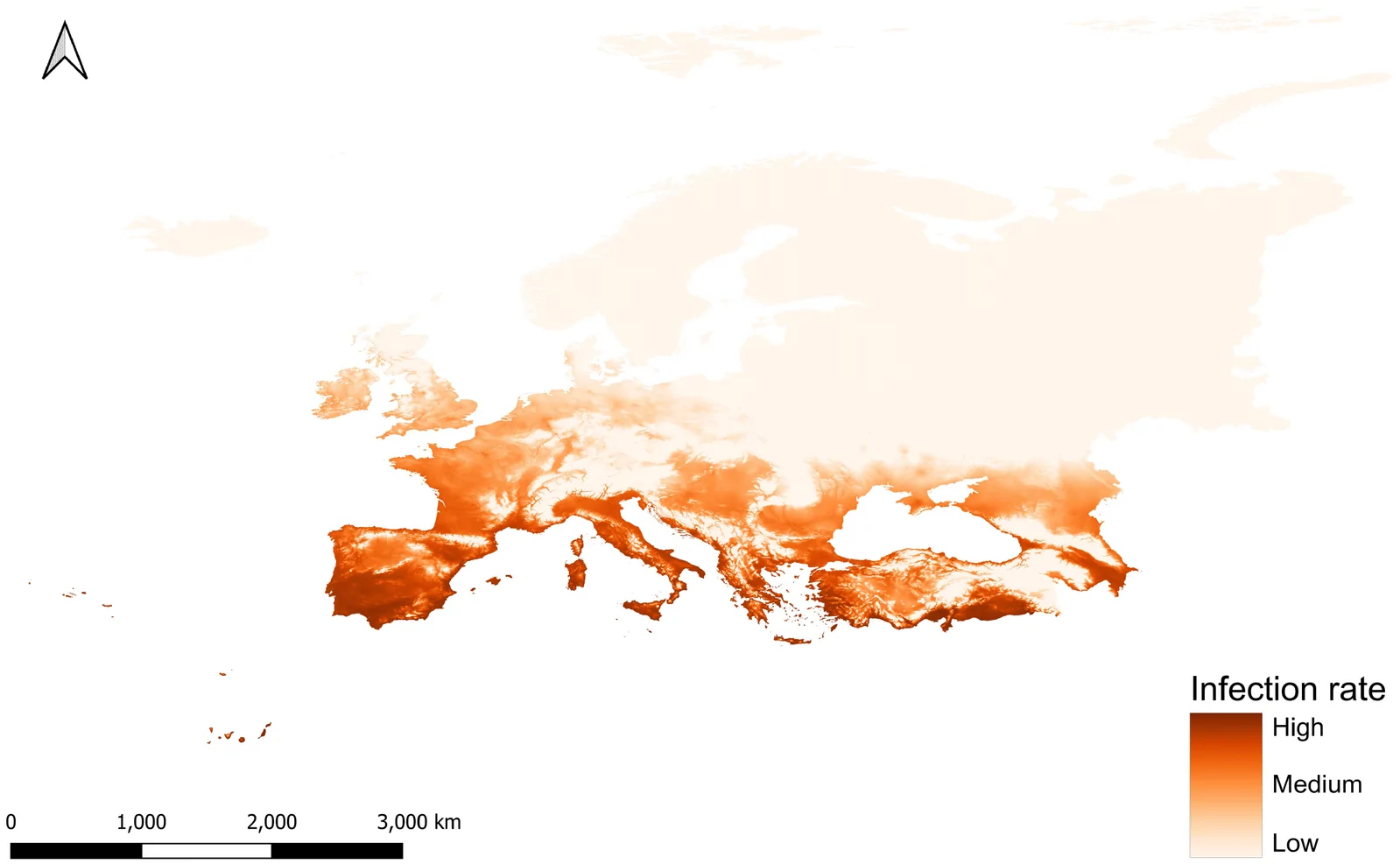
Annual infection rate of *Leishmania infantum* in Europe. Map generated in QGIS (version 3.34.15). Satellite base map: Map data 2026 ESRI. Boundary shapefiles obtained from GADM.

The map representing the *L. infantum* infection rate in the sand fly for the European continent is shown in Fig 2. A color scale ranging from yellow (low infection rate: 0 or 0%), through orange (medium rate: 0.35 or 35%), to red, indicating a high infection rate (0.7 or 70%), has been employed. The highest infection rates are concentrated in the Mediterranean basin: southern and eastern Iberian Peninsula, the Balearic Islands, the Tyrrhenian islands (Corsica, Sardinia, Sicily), southern Italy, and the French Riviera, extending through the Adriatic and Aegean to Turkey and Azerbaijan. The Spanish Northern Plateau, much of Western Europe (including France, Benelux, Ireland, and the southern United Kingdom), the Balkans, and the Black Sea coast present a medium rate. Finally, northern latitudes (Russia, Scandinavia, and Iceland) show low infection rates.

Regarding the monthly infection rate maps (S3 Fig), July showed the highest percentage of infected sand flies, reaching maximum values of up to 0.72 (72%) in Mediterranean countries. Moderate to high values were observed in most of the continent, except for northern latitudes and high-altitude mountain areas, where thermal conditions limit pathogen development. In contrast, winter months (December-February) recorded the lowest infection rates, reaching zero in almost the entire European territory, with the exception of some coastal regions of the Mediterranean where thermal stability allows for the maintenance of residual infectivity values.

### Potential annual infection risk map for leishmaniosis and its validation

**Fig 3:**
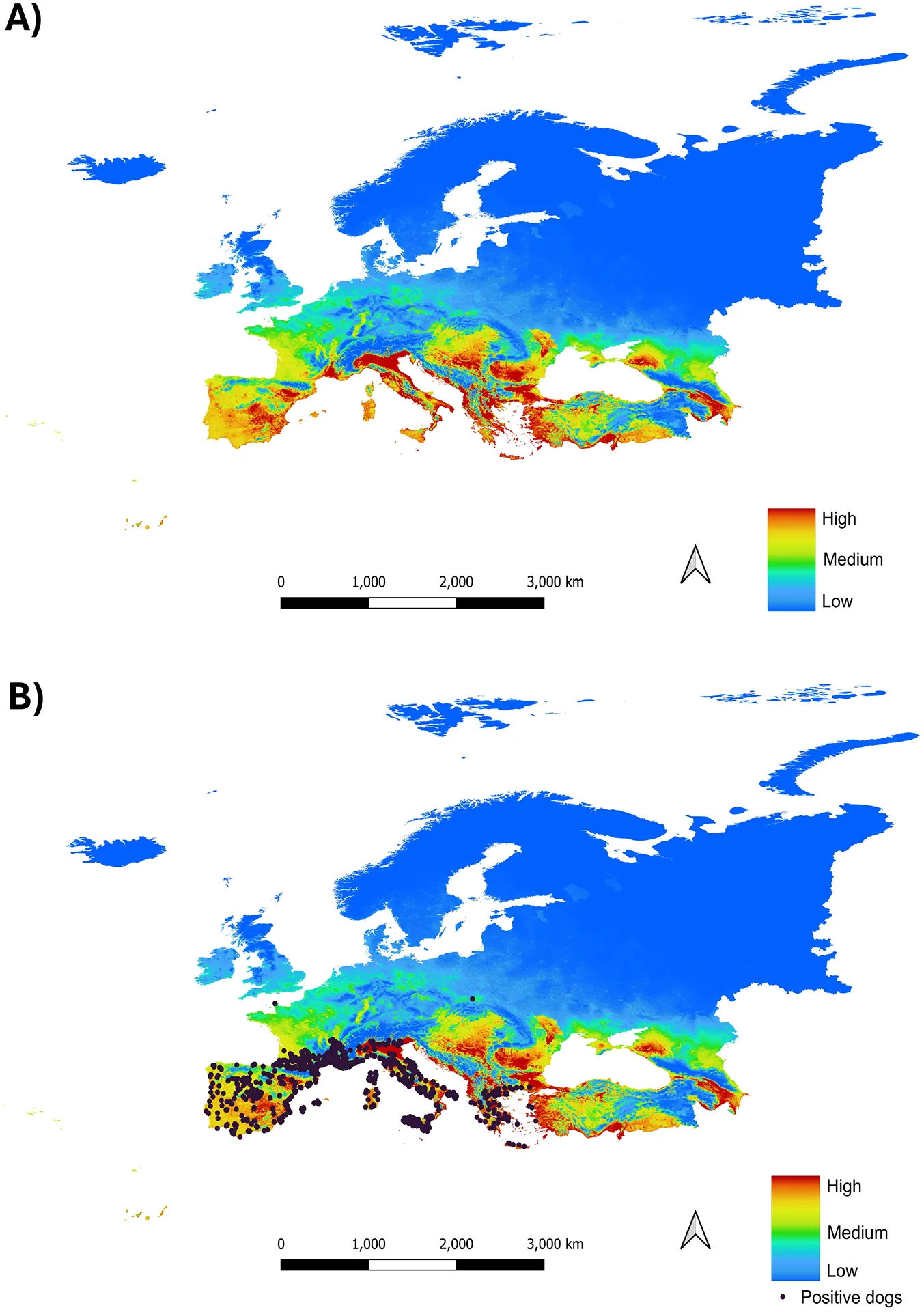
Annual risk map of leishmaniosis infection in Europe (A) and with the georeferenced points of dogs infected with *Leishmania infantum* (B). Map generated in QGIS (version 3.34.15). Satellite base map: Map data 2026 ESRI. Boundary shapefiles obtained from GADM.

After generating potential risk maps for both vectors with different contributions (0–100, 10–90, 25–75, 50–50, 75–25, 90–10, 100-0), weighted with the infection rate, we obtained seven annual *L. infantum* potential infection risk maps in Europe. The risk map with the 50Pn-50Pf contribution (50% *Ph. perniciosus* − 50% *Ph. perfiliewi*) best estimated the percentage of geolocated positive cases in very high/high-risk zones (96.5%) (Table 2, Fig 3). Spatial structure analysis of this risk map showed that the ‘High’ and ‘Very high’ categories occupied only 8.67% and 5.17% of the European territory, respectively, meaning that the high-risk zone was restricted to a mere 13.84% of the total study area. The fact that 96.5% of the 5,315 independent, veterinary-confirmed canine records were concentrated within this limited fraction of the territory confirms the high predictive performance of the model and statistically discounts the likelihood of random spatial overlaps.

**Table 2:** Seven potential risk scenarios generated by combining *Phlebotomus perniciosus* and *Ph. perfiliewi* suitability models at different weighting ratios (0:100, 10:90, 25:75, 50:50, 75:25, 90:10, 100:0), integrated with the estimated parasite infection rate.

Risk	90Pn/10Pf		75Pn/25Pf		50Pn/50Pf		25Pn/75Pf		10Pn/90Pf		100Pn		100Pf	
	Natural Jenks	Positives (%)	Natural Jenks	Positives (%)	Natural Jenks	Positives (%)	Natural Jenks	Positives (%)	Natural Jenks	Positives (%)	Natural Jenks	Positives (%)	Natural Jenks	Positives (%)
Very high	0,769274553-0,452514431	86,10%	0,766765416-0,44201771	85,20%	0,7666049-0,438918884	73,00%	0,782296181-0,429495942	56,60%	0,793111682-0,426103139	47,90%	0,770947278-0,462568367	85,90%	0,802388191-0,421647128	41,80%
High	0,452514431-0,316760102	11%	0,44201771-0,30971309	11,9%	0,438918884-0,309648254	23,5%	0,429495942-0,30064716	38,7%	0,426103139-0,29547298	46,5%	0,462568367-0,323495524	11,3%	0,421647128-0,289489073	52,0%
Medium	0,316760102-0,184022535	2,20%	0,30971309-0,177408469	2,20%	0,309648254-0,180377624	2,80%	0,30064716-0,174866205	4,00%	0,29547298-0,174173546	5,00%	0,323495524-0,187446005	2,20%	0,289489073-0,169917499	5,50%
Low	0,184022535-0,06335202	0,7	0,17740846-0,060138464	0,7	0,180377624-0,060125875	0,70%	0,174866205-0,058288735	0,70%	0,174173546-0,059094596	0,50%	0,187446005-0,063489776	0,60%	0,169917499-0,056639166	0,60%
Very low	0,06335202- 0	0%	0,06013846-0	0%	0,060125875-0	0%	0,058288735-0	0%	0,059094596-0	0,1%	0,063489776-0	0,0%	0,056639166-0	0,1%

The other risk maps showed only modest differences between them, demonstrating the high consistency and robustness of the modeling approach across different weighting combinations.

The selected map presents a higher potential risk of infection in the Po Valley and the Apulia and Lazio regions for Italy, the Levantine coast, the Ebro Depression, and the Southern Plateau in Spain, the French Riviera, the Adriatic coast, the Aegean Sea coast, western and southern Turkey, and countries such as Romania, among others, which coincide with zones characterized by suitable habitats for *Ph. perniciosus* and *Ph. perfiliewi* and a high infection rate. The southeast of the Iberian Peninsula shows a medium-high risk. Meanwhile, northern Spain, eastern France, central-western Turkey, and the Black Sea surroundings present intermediate values, whereas in mountainous areas and the northernmost latitudes (such as Russia, Scandinavia, or the United Kingdom), the risk is apparently low.

### Monthly potential infection risk map for leishmaniosis

**Fig 4:**
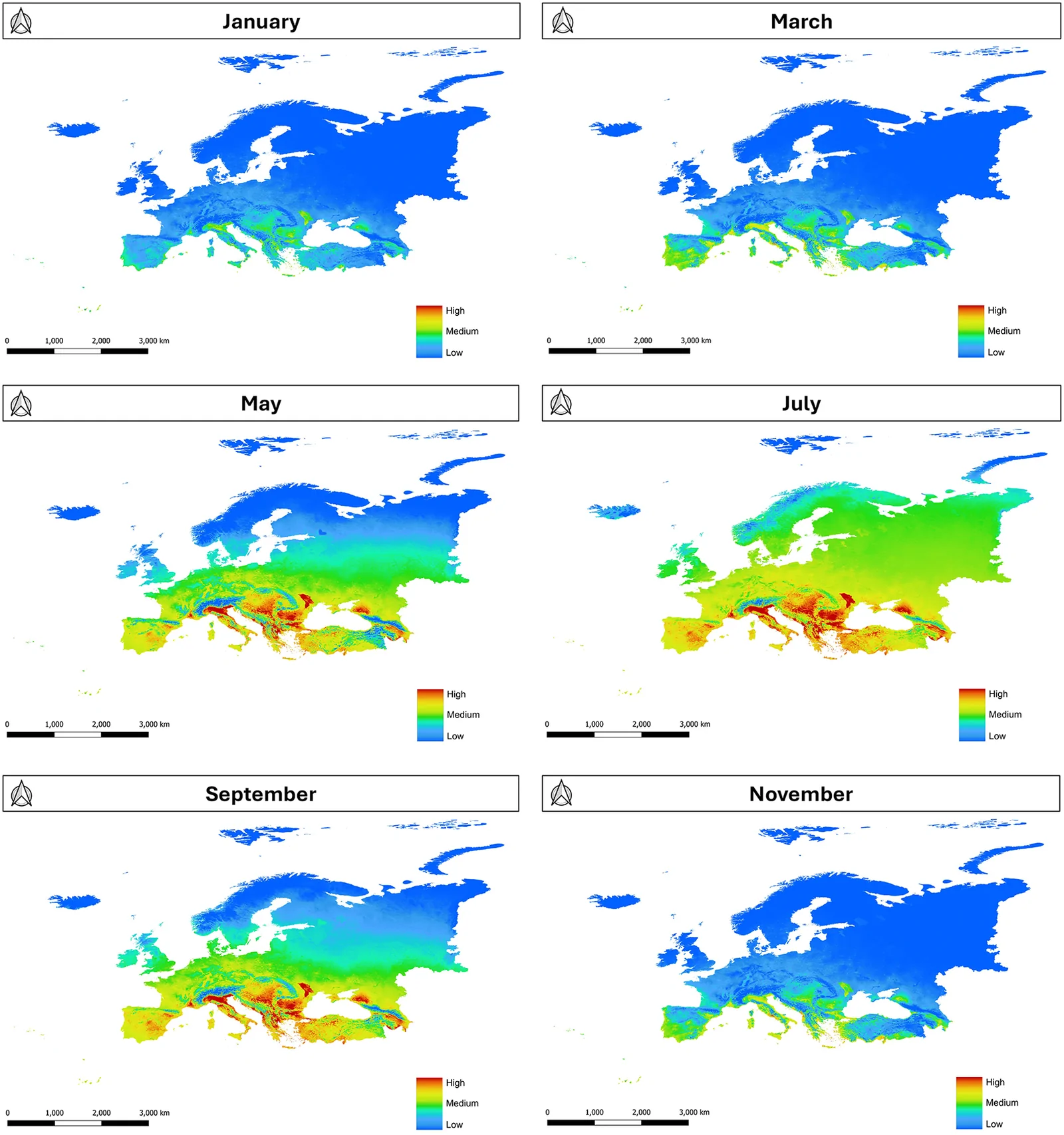
Monthly potential risk map of leishmaniosis infection in Europe each two months (January, March, May, July, September and November). Map generated in QGIS (version 3.34.15). Satellite base map: Map data 2026 ESRI. Boundary shapefiles obtained from GADM.

The analysis of the monthly potential risk maps throughout the year (Fig 4) revealed that, during the winter months (December-February), the infection risk was very low across almost the entire continent, except for some coastal zones of Mediterranean countries (Spain, Portugal, Italy, Greece, and France). In spring (March-May), the infection risk increased gradually from south to north as thermal conditions became more favorable. In summer (June-August), the infection risk increased significantly in most of the continent, except in higher latitudes (northern United Kingdom, northern Russia, and Scandinavia) and high-altitude mountain areas. Southern Europe, due to its warmer climate, showed very high infection risk values, reaching a maximum of 0.7834 in specific zones such as the Po River Valley (Italy) and certain areas of the Balkan Peninsula, while in Central Europe, the risk ranged between moderate and high levels. Finally, during autumn (September-November), the infection risk decreased gradually from north to south, with residual values concentrating once again on the southern continental coast.

### Future projection of potential infection risk

**Fig 5:**
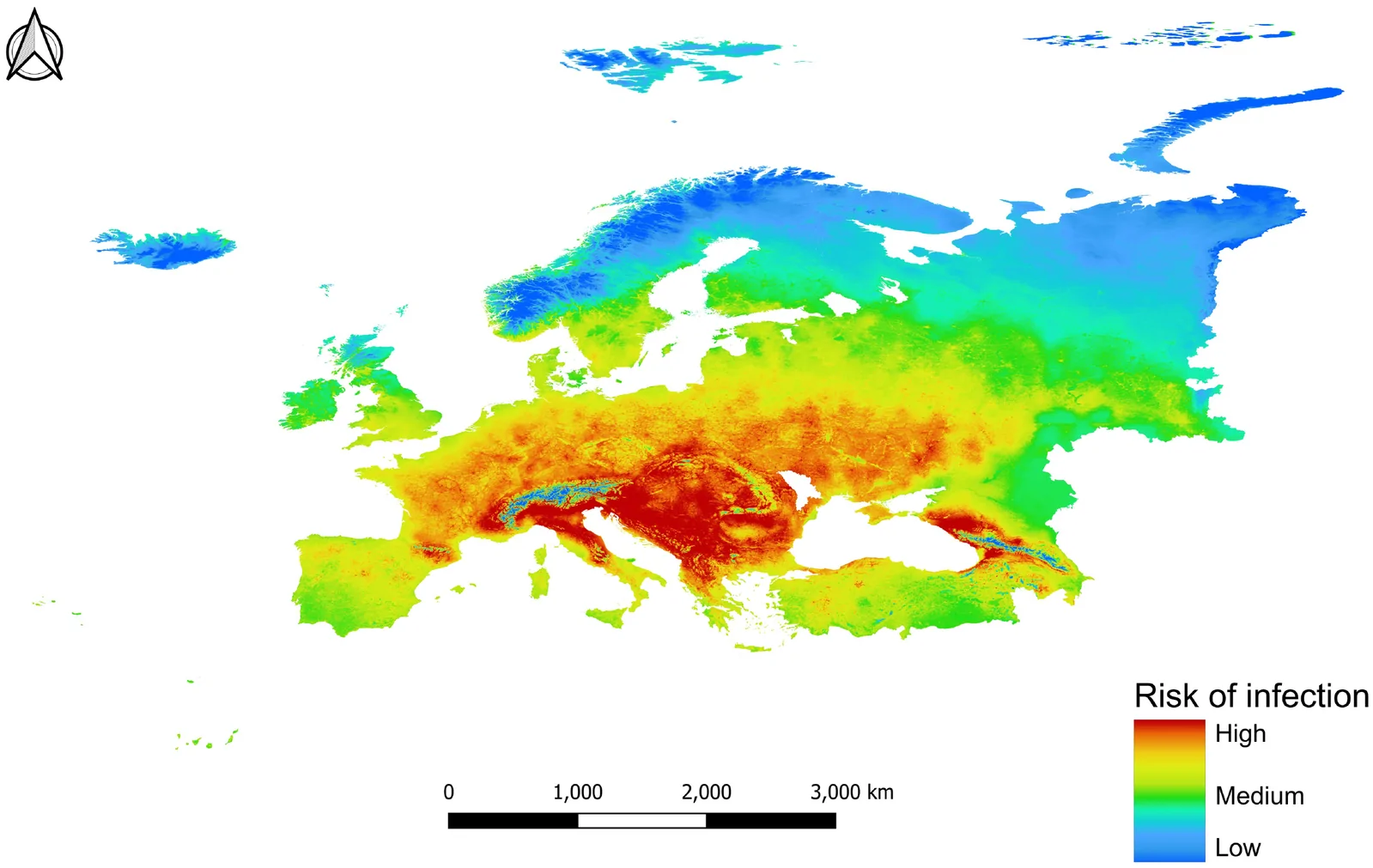
Projection of potential risk map of *Leishmania infantum* for the year 2100 in Europe. Map generated in QGIS (version 3.34.15). Satellite base map: Map data 2026 ESRI. Boundary shapefiles obtained from GADM.

**Fig 6:**
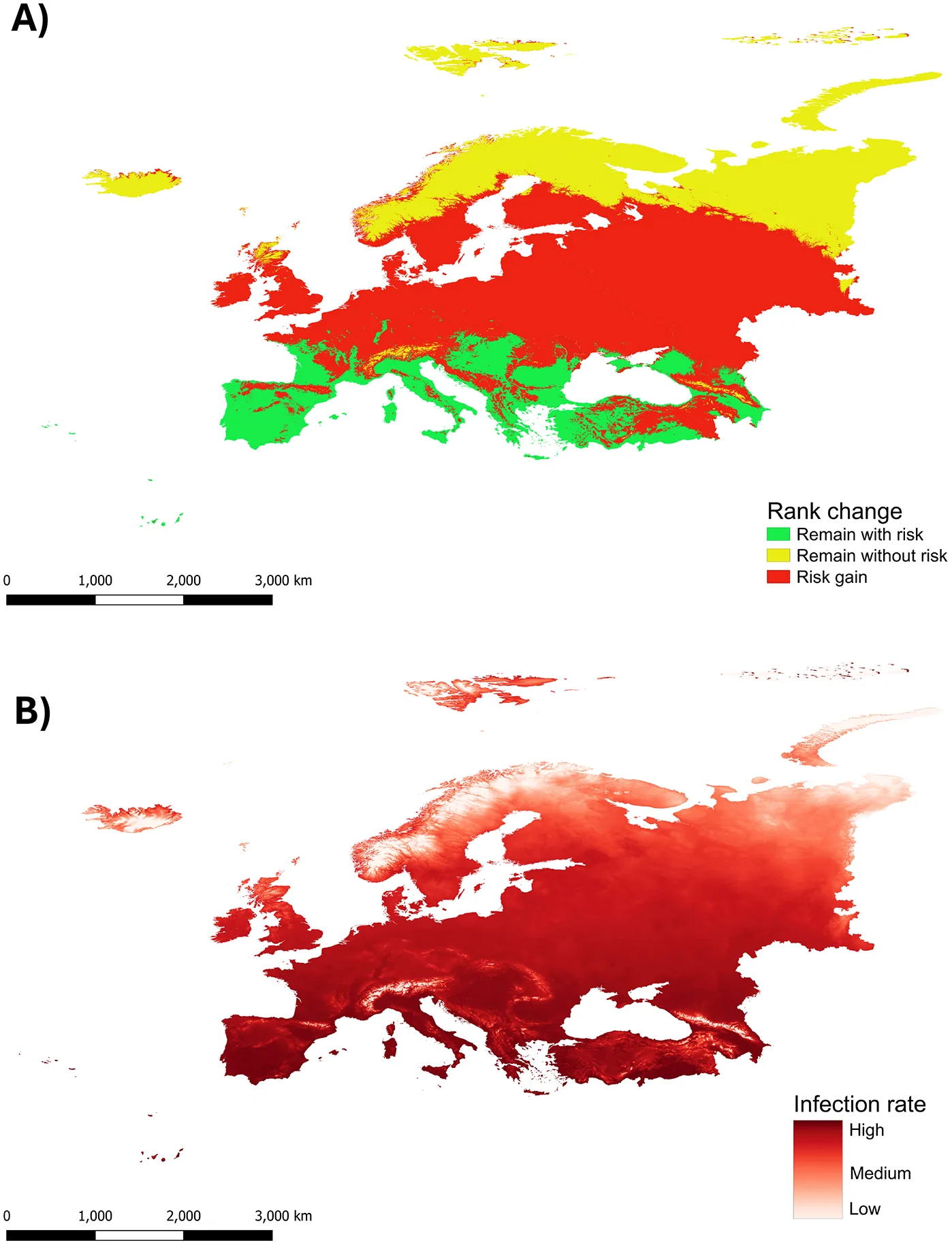
Range change analysis of potential risk infection of *Leishmania infantum* showing areas of gain or remain (A) and infection rate of *Leishmania infantum* (B) for the year 2100 in Europe. Map generated in QGIS (version 3.34.15). Satellite base map: Map data 2026 ESRI. Boundary shapefiles obtained from GADM.

The future projection of the potential risk map for 2100 (Fig 5), which integrates the weighting of both vectors and the projected infection rate for the end of the century, shows a considerable increase in the risk of *L. infantum* infection. The range change analysis indicates an increase of 347.9% at the European territory level, with no areas of loss (0%) recorded (Fig 6A). This increase is mainly located in the northeastern regions of the continent, at higher latitudes, and in higher altitude zones, which currently present cold climates and low suitability for parasite transmission. In the rest of the territories, the infection risk remains stable. Simultaneously, the projected infection rate exhibits a marked expansion towards the north and east of the continent, driven by rising temperatures that favor parasite development in previously limiting latitudes (Fig 6B). Additionally, the seasonal decomposition reveals that while risk in the warmest quarter (BIO_10_) remains geographically restricted to southern regions due to baseline saturation, the coldest quarter (BIO_11_) exhibits a massive spatial expansion across central and eastern Europe (S3 Fig). This is supported by the range change analysis, showing a minor risk gain of 33.1% (0% loss) for BIO_10_, whereas BIO_11_ experiences a substantial risk expansion with a gain of 367,6% and a negligible loss of 0.3%.

## Discussion

This study developed the first *L. infantum* potential infection risk map for the entire European continent, integrating both the habitat suitability of two of its main vectors (*Ph. perniciosus* and *Ph. perfiliewi*) and the estimated parasite infection rate within them, offering a detailed assessment on both an annual and monthly scale. This integrative approach, based on ecological modeling tools such as the MaxEnt algorithm, represents a methodological advance regarding the previously described studies. Unlike approaches restricted to mapping regional vector distribution [15], static climatic risk on an annual scale [6,19], or monthly vector activity using binary models [22], the present study simultaneously integrates environmental factors and parasite biology (infection rate) with continuous temporal resolution. In this way, it overcomes the fragmentation of previous models, offering a tool capable of capturing the complete dynamics of transmission across the European continent.

The habitat suitability models obtained for the vectors *Ph. perniciosus* and *Ph. perfiliewi* showed high predictive capacity, as reflected by the AUC values obtained for each (0.900 and 0.917, respectively), reaching very high discrimination capabilities and falling within the ranges considered optimal for ecological and epidemiological studies (Phillips et al., 2006). The most influential variable was, in both cases, the annual mean temperature (BIO_1_), followed by other temperature-related variables—mean diurnal range (BIO_2_) in the case of *Ph. perniciosus* and temperature seasonality (BIO_4_) in the case of *Ph. perfiliewi*—as the factors contributing most to the model. This corroborates the strong dependence of these vectors on specific climatic conditions such as temperature [36]. This aligns with reports from previous studies in the Mediterranean area [47], where it has been documented that the temperature increase associated with climate change may expand the distribution of these dipterans, favoring the emergence of leishmaniosis in previously risk-free zones.

The inclusion of the pathogen infectivity rate, based on the logarithmic formula proposed by Rioux et al. [36], constitutes one of the main strengths of the present work. The incorporation of this parameter allowed for the estimation not only of the potential distribution of vectors but also of how effective pathogen transmission is as a function of environmental temperature, thus integrating parasitological and entomological components into a single model. Consequently, the resulting maps are not limited to the representation of vector presence but depict potential infection risk maps on both annual and monthly scales, which may have direct utility in veterinary medicine and public health [20,21]. Likewise, the validation procedure using real canine incidence and seroprevalence data and their geographic location provided solidity to the final model. The fact that 96.5% of positive cases were located in areas classified as very high/high risk supports the predictive validity of the potential risk map, positioning it as a useful epidemiological tool for disease surveillance and prevention.

Regarding potential risk distribution, the results obtained identify zones already known for their high endemicity, such as the Mediterranean basin, but also highlight emerging areas such as Romania or Moldova, some regions of southwestern Turkey, and the southern plateau in Spain. It is important to highlight that these zones are characterized by the convergence of high vector habitat suitability and a high estimated infection rate, turning them into transmission hotspots. This information is crucial for establishing intervention priorities, as it allows prevention campaigns, vaccination programs, or vector control strategies to be targeted toward the most exposed areas. On the other hand, the analysis of monthly resolution allows for the dissection of potential risk seasonality, revealing a dynamic of latitudinal expansion and contraction. During the winter months, transmission remains latent, restricted to thermal refuges in the coastal zones of southern Europe. Spring and autumn act as transition periods with risk gradients advancing northward or retreating southward, respectively. It is during the summer that the potential infection risk reaches its maximum geographic extent, covering most of the continent except for boreal latitudes and high mountain zones. The intensity of summer transmission in southern Europe is noteworthy, where high risk peaks were identified in “hotspots” such as the Po River Valley and the Balkan Peninsula, while in Central Europe, the risk ranged between moderate and high levels.

Climate projections for the year 2100 reveal a scenario of dramatic expansion for leishmaniosis in Europe. The models predict a massive increase in habitat suitability for the main vectors, with gains nearing 300% for both *Ph. perniciosus* and *Ph. perfiliewi*. However, the most striking finding lies in the potential risk of *L. infantum* infection, which experiences a net increase of 347.9% without recording areas of risk decrease (0%). This expansion mainly colonizes the northeast of the continent and zones of higher altitude and latitude—areas historically free of the disease due to thermal barriers—while the risk remains stable in current endemic territories. This suggests that climate change will not displace the disease but will expand its geographic range, dismantling the environmental limitations that protected the colder regions of Europe, as observed in other vector-borne diseases [17,48–52].

### Limitations

Despite inherent limitations such as the spatial heterogeneity of the data or the exclusion of socio-sanitary variables [53], this model constitutes a key tool under the One Health approach. Its capacity to identify expansion zones facilitates the implementation of targeted preventive and surveillance measures [54], laying the foundations for the development of dynamic decision-support platforms that can be periodically updated as epidemiological conditions evolve [35].

Furthermore, a limitation of this study is that the multicollinearity analysis and variable selection were conducted using contemporary climate data. While correlations among bioclimatic variables may potentially decouple under future climate scenarios (SSP5-8.5), our projections assume niche conservatism based on current distribution dynamics, meaning that future shifts in variable relationships could introduce uncertainty into the late-century risk maps. These models focus exclusively on environmental predictors and do not account for human and canine demographic changes over the next 75 years. Long-term shifts in population growth, decline, urbanization, or migration patterns could significantly alter host availability and exposure rates, modifying the actual expansion dynamics of the disease.

Additionally, the canine validation dataset is subject to reporting and testing biases, as veterinary surveillance is inherently higher in well-established endemic regions of southern Europe compared to northern territories. While this spatial heterogeneity does not invalidate our validation strategy, readers should interpret the resulting maps with caution when extrapolating risk levels to emerging northern zones.

Finally, this framework focuses exclusively on the domestic canine reservoir, omitting the wildlife cycle. Although dogs are the main epidemiological link to humans in peri-urban areas, wild hosts play a crucial role in maintaining the parasite in natural ecosystems. To fully embrace the proposed One Health approach, future models should incorporate data on wild reservoirs to provide a more comprehensive picture of the risk of transmission.

## Conclusions

The present study has enabled the development, for the first time, of an *L. infantum* potential infection risk map on a continental scale in Europe, integrating the habitat suitability of *Ph. perniciosus* and *Ph. perfiliewi* with the thermal infection rate of the parasite. While the results confirm the persistence of elevated risk in the Mediterranean basin, they indicate an expansion of ecologically suitable areas toward the center and north of the continent, driven by favorable climatic conditions. Consequently, and in light of the increased mobility of animals and humans across Europe and current climatic volatility, it is imperative to transition from static to dynamic models. Therefore, this model constitutes an essential strategic tool under the One Health framework, facilitating the identification of priority areas to optimize surveillance, diagnosis, and sanitary control policies in the face of disease dispersal. From a public and veterinary health perspective, these findings provide actionable data to guide targeted vector-control campaigns and alert medical and veterinary practitioners in non-endemic areas to facilitate early diagnosis and intervention in both human and canine populations. Furthermore, fully achieving this One Health vision will require incorporating transmission dynamics within the sylvatic cycle into future spatial modeling, ensuring that wild reservoirs are integrated into comprehensive, long-term surveillance and control strategies across Europe.

## Supporting information

S1 Fig Presence records of *Phlebotomus perniciosus* (A) and *Ph. perfiliewi* (B) in Europe from 2009 to 2023. Map generated in QGIS (version 3.34.15). Satellite base map: Map data 2026 ESRI. Boundary shapefiles obtained from GADM.
(TIF)

S2 Fig Habitat suitability map for *Phlebotomus perniciosus* (A) and *Ph. perfiliewi* (B) in Europe based on current climatic conditions. Map generated in QGIS (version 3.34.15). Satellite base map: Map data 2026 ESRI. Boundary shapefiles obtained from GADM.
(TIF)

S3 Fig Range change analysis risk map of *Leishmania infantum* for the coldest quarter (A) and for the warmest quarter (B) in 2100 in Europe. Map generated in QGIS (version 3.34.15). Satellite base map: Map data 2026 ESRI. Boundary shapefiles obtained from GADM.
(TIF)

## References

[1] Maia C, Conceição C, Pereira A, Rocha R, Ortuño M, Muñoz C, et al. The estimated distribution of autochthonous leishmaniasis by Leishmania infantum in Europe in 2005-2020. PLoS Negl Trop Dis. 2023;17(7):e0011497. doi: 10.1371/journal.pntd.0011497 37467280 PMC10389729

[2] Tsakmakidis I, Lefkaditis M, Zaralis K, Arsenos G. Alternative hosts of Leishmania infantum: a neglected parasite in Europe. Trop Anim Health Prod. 2024;56(4):128. doi: 10.1007/s11250-024-03978-0 38630347 PMC11189345

[3] Barbero-Moyano J, Gonzálvez M, Bravo-Barriga D, García-Bocanegra I, López-López P, Rivero-Juárez A, et al. Epidemiological landscape in a Mediterranean hotspot of human leishmaniosis in Spain under a One Health approach. Pathog Glob Health. 2025;119(3–4):122–33. doi: 10.1080/20477724.2025.2480083 40122138 PMC12086916

[4] Kaempfle M, Dorsch R, Zablotski Y, Hartmann K, Bergmann M. Importance of Different Parameters for Monitoring Dogs with Leishmania infantum Infections in a Non-Endemic Country. Pathogens. 2025;14(12):1282. doi: 10.3390/pathogens14121282 41471237 PMC12735808

[5] Ferdes I, Latrofa MS, Perles L, Bezerra-Santos MA, Medrouh B, Benmarce M, et al. Occurrence of Leishmania spp. in phlebotomine sand flies and dogs in Guelma region, North-eastern Algeria. Vet Parasitol Reg Stud Reports. 2025;57:101176. doi: 10.1016/j.vprsr.2024.101176 39855864

[6] Carvalho BM, Maia C, Courtenay O, Llabrés-Brustenga A, Lotto Batista M, Moirano G, et al. A climatic suitability indicator to support Leishmania infantum surveillance in Europe: a modelling study. Lancet Reg Health Eur. 2024;43:100971. doi: 10.1016/j.lanepe.2024.100971 39040529 PMC11261136

[7] Maia C. Sand fly-borne diseases in Europe: epidemiological overview and potential triggers for their emergence and re-emergence. J Comp Pathol. 2024;209:6–12. doi: 10.1016/j.jcpa.2024.01.001 38320331

[8] Plesko A, Florea T, Imre M, Hoffman D, Dreghiciu IC, Pocinoc A, et al. Canine Leishmaniasis in Europe over the Last Decade: A Review of Geographic Trends and Epidemiological Data. Pathogens. 2025;14(11):1082. doi: 10.3390/pathogens14111082 41305320 PMC12655625

[9] Fooks AR, Johnson N. Jet set pets: examining the zoonosis risk in animal import and travel across the European Union. Vet Med (Auckl). 2014;6:17–25. doi: 10.2147/VMRR.S62059 30101093 PMC6067792

[10] de Koeijer A, Fischer EAJ. Calculating the probability of introduction of Leishmania infantum into the Netherlands through import of dogs. Prev Vet Med. 2017;142:13–9.

[11] Romanello M, Walawender M, Hsu S-C, Moskeland A, Palmeiro-Silva Y, Scamman D, et al. The 2024 report of the Lancet Countdown on health and climate change: facing record-breaking threats from delayed action. Lancet. 2024;404(10465):1847–96. doi: 10.1016/S0140-6736(24)01822-1 39488222 PMC7616816

[12] Ravasi D, Schnyder M, Guidi V, Haye T, Parrondo Monton D, Flacio E. Exploring Emerging Challenges: Survey on Phlebotomine Sand Flies and Leishmania infantum at the Northern Endemic Border in Europe. Pathogens. 2024;13(12):1074. doi: 10.3390/pathogens13121074 39770334 PMC11728847

[13] Hanafi-Bojd AA, Yaghoobi-Ershadi MR, Haghdoost AA, Akhavan AA, Rassi Y, Karimi A, et al. Modeling the Distribution of Cutaneous Leishmaniasis Vectors (Psychodidae: Phlebotominae) in Iran: A Potential Transmission in Disease Prone Areas. J Med Entomol. 2015;52(4):557–65. doi: 10.1093/jme/tjv058 26335462

[14] Hanafi-Bojd AA, Rassi Y, Yaghoobi-Ershadi MR, Haghdoost AA, Akhavan AA, Charrahy Z, et al. Predicted Distribution of Visceral Leishmaniasis Vectors (Diptera: Psychodidae; Phlebotominae) in Iran: A Niche Model Study. Zoonoses Public Health. 2015;62(8):644–54. doi: 10.1111/zph.12202 26032232

[15] Sofizadeh A, Rassi Y, Vatandoost H, Hanafi-Bojd AA, Mollalo A, Rafizadeh S, et al. Predicting the Distribution of Phlebotomus papatasi (Diptera: Psychodidae), the Primary Vector of Zoonotic Cutaneous Leishmaniasis, in Golestan Province of Iran Using Ecological Niche Modeling: Comparison of MaxEnt and GARP Models. J Med Entomol. 2017;54(2):312–20. doi: 10.1093/jme/tjw178 28025245

[16] Falcão de Oliveira E, Galati EAB, de Oliveira AG, Rangel EF, de Carvalho BM. Ecological niche modelling and predicted geographic distribution of Lutzomyia cruzi, vector of Leishmania infantum in South America. PLoS Negl Trop Dis. 2018;12(7):e0006684. doi: 10.1371/journal.pntd.0006684 30059494 PMC6085070

[17] Charrahy Z, Yaghoobi-Ershadi MR, Shirzadi MR, Akhavan AA, Rassi Y, Hosseini SZ, et al. Climate change and its effect on the vulnerability to zoonotic cutaneous leishmaniasis in Iran. Transbound Emerg Dis. 2022;69(3):1506–20. doi: 10.1111/tbed.14115 33876891

[18] Acharya BK, Vegvari C, Lillywhite J, Lillywhite HJ, Kloos D, Sharma N, et al. Climate change and its impact on spatial and temporal distribution of visceral leishmaniasis transmission risk in Nepal. BMC Infect Dis. 2025;25(1):1303. doi: 10.1186/s12879-025-11757-x 41088037 PMC12523026

[19] Cunze S, Kochmann J, Koch LK, Hasselmann KJQ, Klimpel S. Leishmaniasis in Eurasia and Africa: geographical distribution of vector species and pathogens. R Soc Open Sci. 2019;6(5):190334. doi: 10.1098/rsos.190334 31218068 PMC6549972

[20] Rodríguez-Escolar I, Balmori-de la Puente A, Collado-Cuadrado M, Bravo-Barriga D, Delacour-Estrella S, Hernández-Lambraño RE, et al. Analysis of the current risk of Leishmania infantum transmission for domestic dogs in Spain and Portugal and its future projection in climate change scenarios. Front Vet Sci. 2024;11:1399772. doi: 10.3389/fvets.2024.1399772 38756515 PMC11096601

[21] Rodríguez-Escolar I, Morchón R, Papadopoulos E, Sioutas G, Collado-Cuadrado M, Infante González-Mohino E, et al. Analysis of the Current Risk of Leishmania infantum Transmission in Greece and its Projection. Transbound Emerg Dis. 2025;2025:1087533. doi: 10.1155/tbed/1087533 40692873 PMC12277051

[22] Bozorg-Omid F, Youssefi F, Hassanpour G, Rahimi Foroushani A, Rahimi M, Shirzadi MR, et al. Predicting the Effect of Temperature Changes on Phlebotomus papatasi Activity, as the Main Vector of Zoonotic Cutaneous Leishmaniasis in Iran. Transbound Emerg Dis. 2025;2025:9518371. doi: 10.1155/tbed/9518371 40302736 PMC12017177

[23] Berentsen WH, Poulsen TM, Windley BF, East WG. Europe. Encyclopedia Britannica. 2025. https://www.britannica.com/place/Europe

[24] Antonescu B, Mărmureanu L, Vasilescu J, Marin C, Andrei S, Boldeanu M. A 41‐year bioclimatology of thermal stress in Europe. Int J Climatol. 2021;41(7):3934–52.

[25] Alten B, Maia C, Afonso MO, Campino L, Jiménez M, González E, et al. Seasonal Dynamics of Phlebotomine Sand Fly Species Proven Vectors of Mediterranean Leishmaniasis Caused by Leishmania infantum. PLoS Negl Trop Dis. 2016;10(2):e0004458. doi: 10.1371/journal.pntd.0004458 26900688 PMC4762948

[26] European Centre for Disease Prevention and Control. Phlebotomine sandfly maps. 2023. https://www.ecdc.europa.eu/en/disease-vectors/surveillance-and-disease-data/phlebotomine-maps

[27] European Centre for Disease Prevention and Control, European Food Safety Authority. VectorNet: A European network for sharing data on the geographic distribution of arthropod vectors, transmitting human and animal disease agents. Stockholm: ECDC/EFSA. 2025.

[28] Ivović V, Patakakis M, Tselentis Y, Chaniotis B. Faunistic study of sandflies in Greece. Med Vet Entomol. 2007;21(1):121–4. doi: 10.1111/j.1365-2915.2006.00649.x 17373955

[29] Fick SE, Hijmans RJ. WorldClim 2: new 1‐km spatial resolution climate surfaces for global land areas. Int J Climatol. 2017;37(12):4302–15.

[30] R Core Team. R: A language and environment for statistical computing. 2023. https://www.R-project.org/

[31] EarthEnv. 2014. https://www.earthenv.org/

[32] Socioeconomic Data and Applications Center. 2018. https://search.earthdata.nasa.gov/

[33] Phillips SJ, Anderson RP, Schapire RE. Maximum entropy modeling of species geographic distributions. Ecol Model. 2006;190(3–4):231–59.

[34] Cobos ME, Peterson AT, Barve N, Osorio-Olvera L. kuenm: an R package for detailed development of ecological niche models using Maxent. PeerJ. 2019;7:e6281. doi: 10.7717/peerj.6281 30755826 PMC6368831

[35] Balmori-de la Puente A, Rodríguez-Escolar I, Collado-Cuadrado M, Infante González-Mohino E, Vieira Lista MC, Hernández-Lambraño RE, et al. Transmission risk of vector-borne bacterial diseases (Anaplasma spp. and Ehrlichia canis) in Spain and Portugal. BMC Vet Res. 2024;20(1):526. doi: 10.1186/s12917-024-04383-3 39593089 PMC11590303

[36] Rioux JA, Aboulker JP, Lanotte G, Killick-Kendrick R, Martini-Dumas A. Écologie des leishmanioses dans le sud de la France-21—Influence de la température sur le développement de Leishmania infantum Nicolle, 1908 chez Phlebotomus ariasi Tonnoir, 1921. Étude expérimentale. Ann Parasitol Hum Comp. 1985;60(3):221–9. 4062175 10.1051/parasite/1985603221

[37] CHELSA Climate. Climatologies at high resolution for the earth’s land surface areas. 2026. https://chelsa-climate.org/10.1038/sdata.2017.122 PMC5584396 28872642

[38] MyVBDmap. Ceva Santé Animale. 2025. https://www.myvbdmap.com

[39] Symeonidou I, Angelou A, Theodoridis A, Sioutas G, Papadopoulos E. Canine Leishmaniosis in Greece: An Updated Countrywide Serological Study and Associated Risk Factors. Pathogens. 2021;10(9):1129. doi: 10.3390/pathogens10091129 34578159 PMC8470449

[40] Almeida M, Maia C, Cristóvão JM, Morgado C, Barbosa I, Ibars RF, et al. Seroprevalence and Risk Factors Associated with Leishmania Infection in Dogs from Portugal. Microorganisms. 2022;10(11):2262. doi: 10.3390/microorganisms10112262 36422332 PMC9695918

[41] Montoya-Alonso JA, Morchón R, Costa-Rodríguez N, Matos JI, Falcón-Cordón Y, Carretón E. Current Distribution of Selected Vector-Borne Diseases in Dogs in Spain. Front Vet Sci. 2020;7:564429. doi: 10.3389/fvets.2020.564429 33195540 PMC7643126

[42] Andrews MB, Ridley JK, Wood RA, Andrews T, Belcher SE, Bushell AC. Historical simulations with HadGEM3‐GC3. 1 for CMIP6. J Adv Model Earth Syst. 2020;12(6):e2019MS001995. doi: 10.1029/2019MS001995

[43] Kuhlbrodt T, Jones CG, Sellar A, Storkey D, Blockley E, Stringer M, et al. The low‐resolution version of HadGEM3 GC3.1: Development and evaluation for global climate. J Adv Model Earth Syst. 2018;10(11):2865–88. 30774751 10.1029/2018MS001370 PMC6360456

[44] Zelinka MD, Myers TA, McCoy DT, Po‐Chedley S, Caldwell PM, Ceppi P. Geophys Res Lett. 2020;47(1):e2019GL085782. doi: 10.1029/2019GL085782

[45] Palmer TE, McSweeney CF, Booth BBB, Priestley MDK, Davini P, Brunner L, et al. Performance-based sub-selection of CMIP6 models for impact assessments in Europe. Earth Syst Dynam. 2023;14(2):457–83. doi: 10.5194/esd-14-457-2023

[46] Thuiller W, Lafourcade B, Engler R, Araújo MB. BIOMOD – a platform for ensemble forecasting of species distributions. Ecography. 2009;32(3):369–73. doi: 10.1111/j.1600-0587.2008.05742.x

[47] Chalghaf B, Chemkhi J, Mayala B, Harrabi M, Benie GB, Michael E, et al. Ecological niche modeling predicting the potential distribution of Leishmania vectors in the Mediterranean basin: impact of climate change. Parasit Vectors. 2018;11(1):461. doi: 10.1186/s13071-018-3019-x 30092826 PMC6085715

[48] Githeko AK, Lindsay SW, Confalonieri UE, Patz JA. Climate change and vector-borne diseases: a regional analysis. Bull World Health Organ. 2000;78(9):1136–47. 11019462 PMC2560843

[49] Semenza JC, Menne B. Climate change and infectious diseases in Europe. Lancet Infect Dis. 2009;9(6):365–75. doi: 10.1016/S1473-3099(09)70104-5 19467476

[50] Hongoh V, Berrang-Ford L, Scott ME, Lindsay LR. Expanding geographical distribution of the mosquito, Culex pipiens, in Canada under climate change. Appl Geogr. 2012;33:53–62.

[51] Kartashev V, Afonin A, González-Miguel J, Sepúlveda R, Simón L, Morchón R, et al. Regional warming and emerging vector-borne zoonotic dirofilariosis in the Russian Federation, Ukraine, and other post-Soviet states from 1981 to 2011 and projection by 2030. Biomed Res Int. 2014;2014:858936. doi: 10.1155/2014/858936 25045709 PMC4090463

[52] Hanafi-Bojd AA, Vatandoost H, Yaghoobi-Ershadi MR. Climate Change and the Risk of Malaria Transmission in Iran. J Med Entomol. 2020;57(1):50–64. doi: 10.1093/jme/tjz131 31429469

[53] Vilas-Boas DF, Nakasone EKN, Gonçalves AAM, Lair DF, de Oliveira DS, Pereira DFS, et al. Global Distribution of Canine Visceral Leishmaniasis and the Role of the Dog in the Epidemiology of the Disease. Pathogens. 2024;13(6):455. doi: 10.3390/pathogens13060455 38921753 PMC11206782

[54] Cosma C, Maia C, Khan N, Infantino M, Del Riccio M. Leishmaniasis in Humans and Animals: A One Health Approach for Surveillance, Prevention and Control in a Changing World. Trop Med Infect Dis. 2024;9(11):258. doi: 10.3390/tropicalmed9110258 39591264 PMC11598728

